# Effects of Long-Term Exposure to 60 GHz Millimeter-Wavelength Radiation on the Genotoxicity and Heat Shock Protein (Hsp) Expression of Cells Derived from Human Eye

**DOI:** 10.3390/ijerph13080802

**Published:** 2016-08-08

**Authors:** Shin Koyama, Eijiro Narita, Yoko Shimizu, Yukihisa Suzuki, Takeo Shiina, Masao Taki, Naoki Shinohara, Junji Miyakoshi

**Affiliations:** 1 Laboratory of Applied Radio Engineering for Humanosphere, Research Institute for Sustainable Humanosphere, Kyoto University, Uji, Kyoto 611-0011, Japan; narita.eijirou.4e@kyoto-u.ac.jp (E.N.); yoko_shimizu@rish.kyoto-u.ac.jp (Y.S.); shino@rish.kyoto-u.ac.jp (N.S.); miyakoshi@rish.kyoto-u.ac.jp (J.M.); 2 Department of Electrical & Electronic Engineering, Graduate Schools of Science and Engineering, Tokyo Metropolitan University, 1-1, Hachioji, Tokyo 192-0397, Japan; y_suzuki@tmu.ac.jp (Y.S.); shiina-takeo@ed.tmu.ac.jp (T.S.); masao@tmu.ac.jp (M.T.)

**Keywords:** millimeter-waves, cellular genotoxicity, micronucleus formation, comet assay, heat shock protein, long-term exposure, human eye cells

## Abstract

Human corneal epithelial (HCE-T) and human lens epithelial (SRA01/04) cells derived from the human eye were exposed to 60 gigahertz (GHz) millimeter-wavelength radiation for 24 h. There was no statistically significant increase in the micronucleus (MN) frequency in cells exposed to 60 GHz millimeter-wavelength radiation at 1 mW/cm^2^ compared with sham-exposed controls and incubator controls. The MN frequency of cells treated with bleomycin for 1 h provided positive controls. The comet assay, used to detect DNA strand breaks, and heat shock protein (Hsp) expression also showed no statistically significant effects of exposure. These results indicate that exposure to millimeter-wavelength radiation has no effect on genotoxicity in human eye cells.

## 1. Introduction

The past decade has seen rapid changes in the technology used to communicate electronically. New communications technologies will undoubtedly arise and spread despite limitations in available radio frequencies. There is, therefore, demand for access to new frequency ranges of the electromagnetic spectrum. However, the rapid introduction of wireless devices has increased the use of millimeter-wavelength (30–300 gigahertz (GHz)) technologies, and with it increased public concern about possible adverse effects of radiation sources on human health. It was previously reported that wide-band millimeter-wavelength exposure at 53–78 GHz significantly inhibited the proliferation of human skin melanoma cells [1]. In addition, Korenstein-Ilan et al. [2] reported that 0.1 terahertz (THz) continuous-wave radiation increased the genomic instability of human lymphocytes. Another study indicated that expression of the adiponectin, GLUT4 and PPARG genes showed clear effects of THz irradiation after prolonged, broad-band exposure of mesenchymal mouse stem cells [3]. A recent study reported that THz radiation induced spindle disturbances in human-hamster hybrid cells [4], and other studies also reported negative physiological effects [5,6,7]. However, there are also several reports showing some non-thermal effects on biological membranes from millimeter-wavelength exposure [8,9,10,11,12]. It is therefore necessary to evaluate the influence of low-level exposure of the human body to millimeter-wavelengths because the energy of millimeter-wavelength radiation is absorbed by the body’s surface, in particular the skin and eyes [13,14]. To investigate the non-thermal effects of millimeter-wavelength radiation, we developed a device to expose cells to 60 GHz millimeter-waves and assessed the frequency of micronucleus (MN) formation, single-strand breaks in the DNA, and the expression of heat shock proteins (Hsp) in cells derived from the human eye.

## 2. Materials and Methods

### 2.1. Millimeter-Wave Exposure Set-Up

**Figure 1:**
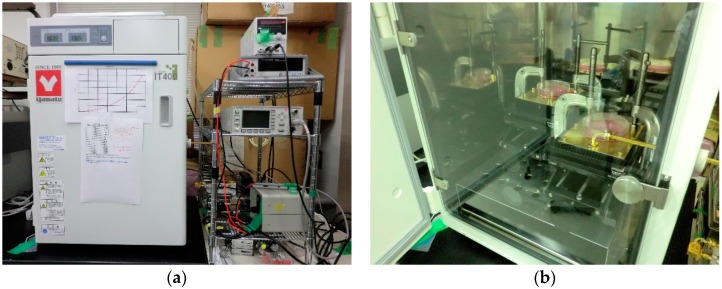
The built-in incubator of the 60 GHz exposure system (**a**) and an inside view of the incubator (**b**). The chamber of the exposure system was maintained under controlled conditions similar to those in an incubator, i.e., an atmosphere of 95% air and 5% CO_2_ at a relative humidity of >95% and a temperature of 37 °C.

We used a specially designed apparatus to expose cells to 60 GHz millimeter-waves (Figure 1a,b). Details of the exposure system were described previously [15]. Briefly, the exposure conditions were as follows. The chamber in the exposure system was maintained under controlled conditions similar to those in a conventional incubator, i.e., an atmosphere of 95% air and 5% CO_2_ at a relative humidity of >95% and a temperature of 37 ± 0.1 °C. The exposure apparatus was based on a printed circuit board with a disc-shaped area surrounded by a post-wall waveguide to feed millimeter-waves into the substrate of the exposure area. A culture dish 100 mm in diameter was placed on the disc-shaped area of the apparatus and 60 GHz millimeter-waves were applied to the bottom of the dish through narrow slot coupling windows on the top of the circuit board, thus exposing cells adhered to the bottom of the culture dish to millimeter-waves. The spatially averaged power density was set 1 mW/cm^2^ at the bottom of the culture medium and temperature elevation was maintained to less than 0.1 °C. 

### 2.2. Cell Culture

The HCE-T human corneal epithelial cell line (RIKEN CELL BANK, Ibaraki, Japan) derived from human corneal epithelial cells was maintained in DMEM (Wako Pure Chemical Industries, Ltd., Osaka, Japan):HamF12 (Wako Pure Chemical Industries, Ltd.) (1:1) medium supplemented with 5% fetal bovine serum (FBS), insulin (Sigma-Aldrich, St. Louis, MO, USA) at a final concentration of 5 μg/mL, and human epidermal growth factor (Roche, Basel, Switzerland) at a final concentration of 10 ng/mL. This cell line was kindly supplied by Masao Taki, Tokyo Metropolitan University. The SRA01/04 human lens epithelial cell line (RIKEN CELL BANK) derived from human lens epithelial cells was maintained in DMEM (Wako Pure Chemical Industries, Ltd.) medium supplemented with 20% FBS. This cell line was kindly supplied by Hiroshi Sasaki, Kanazawa Medical University. The cells were seeded onto 10 cm dishes (Asahi Glass, Tokyo, Japan) at a density of 1 × 10^6^ cells/mL with total volume of 11.6 mL medium to adjust the medium layer thickness to 2 mm for ideal exposure. After overnight culturing, the cells were exposed to 60 GHz. The cells were collected 24 h after exposure to millimeter-wavelength radiation. For positive controls, cells were treated with 10 μg/mL bleomycin for 1 h for the genotoxicity test and with heat (43 °C for 2 h and then 37 °C for 1 or 2 h) for the Hsp expression test.

### 2.3. Micronucleus (MN) Frequency

The methodology for conducting the Micronucleus (MN) formation test was described previously [16]. Briefly, after exposure to millimeter-wavelength radiation or 10 μg/mL bleomycin for 1 h, the cells were cultured in medium supplemented with 3 μg/mL cytochalasin B (Sigma-Aldrich) in a conventional incubator for 24 h, centrifuged onto slides using a Cytospin centrifuge (Shandon Southern Instruments Ltd., Cambridge, UK) at 100× *g* for 5 min, fixed with cold 80% ethanol for 30 min, and stained with 0.2 μg/mL propidium iodide (Sigma-Aldrich). A total of 1000 binucleated cells were counted and the frequency of MN formation was determined using a fluorescence microscope (Olympus, Tokyo, Japan) according to the criteria described previously [17]. At least three independent tests were performed.

### 2.4. Comet Assay

The comet assay was performed using a Trevigen Comet Assay Kit (Trevigen Inc., Gaithersburg, MD, USA) to detect DNA strand breaks at the single-cell level as described previously [18]. Briefly, cells were exposed to millimeter-wavelength radiation for 24 h, collected by trypsinization and centrifuged immediately after exposure, then mixed with low melting point agarose to prepare a cell suspension in 0.1% agarose/phosphate buffered saline (PBS). After gelation of the agarose, the cells were lysed, then the alkaline unwinding was performed (1 h at 4 °C, pH > 13). The resulting DNA samples were electrophoresed at 1 V/cm for 30 min in a 0.3 M NaOH (Nacalai Tesque, Kyoto, Japan) and 1 mM ethylenediamine-*N*,*N*,*N*’,*N*’-tetraacetic acid (Sigma-Aldrich) solution. After the DNA was stained with SYBR Green I, immunofluorescence images were captured using a fluorescence microscope (Olympus, Tokyo, Japan). DNA strand breaks were analyzed using Comet software (Perceptive Instruments, Suffolk, UK). At least 100 comets from each gel were analyzed, and at least five independent experiments were performed. Tail length indicates the pixel length of the comet tail, tail percent indicates the percentage of tail content relative to comet content, and tail moment was calculated as follows:

Tail moment = (the distance between the center of the comet head and the center of the comet tail) × (tail percent)/100(1)

### 2.5. Hsp Expression

The methodology for quantifying Hsp expression has been described previously [18]. Briefly, after millimeter-wavelength exposure or heat treatment, the cells were washed with cold PBS, collected using a cell scraper, and the proteins were extracted using CelLyticTM-M (Sigma-Aldrich) supplemented protease inhibitor cocktail (Sigma-Aldrich). The extracted proteins were incubated at 4 °C for 15 min and centrifuged at 100× g for 15 min. The supernatants were collected and the protein concentrations were measured using an iMark plate reader (Bio-Rad, Hercules, CA, USA) and a calibration curve and adjusted to 1 mg/mL. The samples were mixed with 2ME sample buffer (Wako) at a ratio of 1:1 and incubated at 100 °C for 1 min, then immediately put on ice. Extracted protein (20 µg) was loaded onto a 12.5% sodium dodecyl sulfate (SDS)-polyacrylamide gel (Wako), separated by electrophoresis, and transferred to a nitrocellulose membrane (Life Technologies Japan, Tokyo, Japan) using iBlot (Life Technologies Japan). BenchProTM 4100 (Invitrogen, Carlsbad, CA, USA) was used for blocking and immunostaining. The membrane was blocked with skim milk (DS Farma Biomedical, Osaka, Japan) for 1 h and immunostained with antibodies for 1 h. Primary antibody for Hsp27 (R&D Systems, Minneapolis, MN, USA, 1:10,000), Hsp70 (StressMarq Biosciences Inc., Victoria, BC, Canada, 1:1000), Hsp90α (StressMarq, 1:2000) and β-actin (Sigma-Aldrich, 1:1000) were used. The secondary antibodies used were anti-mouse (GE Healthcare, Tokyo, Japan, 1:1000), anti-goat (R&D, 1:500) and anti-rabbit (Sigma-Aldrich, 1:500). After immunostaining, the membranes were stained with horseradish peroxidase, followed by analysis using ATTO Image Analysis Software (Tokyo, Japan). At least three independent experiments were performed.

### 2.6. Statistical Analysis

The data were analyzed using Tukey’s test. *p*-Values < 0.05 or 0.01 were considered to be statistically significant. The statistical power (1−β) in MN and comet assay test were calculated using the effect size (*f*) = 0.1. All assays were performed in a blinded fashion.

## 3. Results

### 3.1. MN Formation

**Figure 2:**
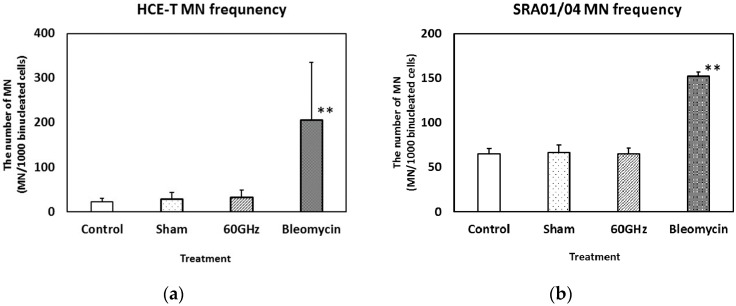
Micronucleus frequency in cells exposed to millimeter-wavelength radiation at 60 GHz for 24 h: HCE-T cells (**a**) and SRA01/04 cells (**b**). Treatment with bleomycin (10 µg/mL) provided the positive controls. Data are presented as the mean ± SD from three independent experiments. Asterisks ** indicate *p* < 0.01. *f* = 0.1, (1−β) = 0.9903.

The frequency of MN formation in HCE-T and SRA01/04 cells is shown in Figure 2a,b, respectively. MN frequency in HCE-T and SRA01/04 cells increased significantly following bleomycin treatment, whereas no significant difference was observed between incubator control, sham-exposure, and the millimeter-wavelength–exposed cells. These results suggest that 24 h exposure to 60 GHz millimeter-wavelength radiation may have no significant effect on the MN frequency in HCE-T and SRA01/04 cells.

### 3.2. Comet Assay

**Figure 3:**
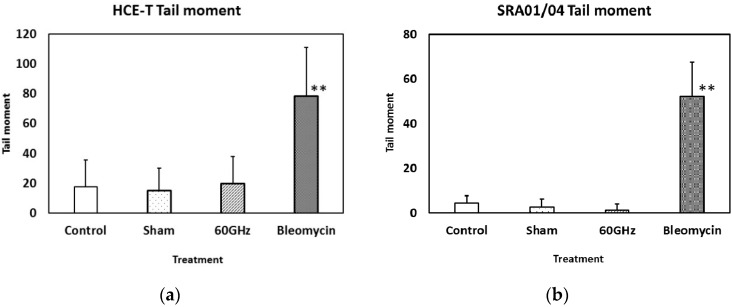
Values of a comet parameter (tail moment) for cells exposed to millimeter-wavelength 60 GHz radiation for 24 h: HCE-T (**a**) and SRA01/04 (**b**). The positive control was treatment with bleomycin (10 µg/mL). Data are presented as the mean ± SD from three independent experiments. Asterisks ** indicate *p* < 0.01. *f* = 0.1, (1−β) = 0.2193.

The tail moments of lysed HCE-T and SRA01/04 cells are shown in Figure 3a,b, respectively. The tail moment indicates the degree of the genotoxic effect on the DNA. The tail moments of both HCE-T and SRA01/04 cells increased significantly following the bleomycin treatment, whereas no significant difference in the tail moment was observed between the incubator control, sham and millimeter-wave exposure samples. The tail moments were calculated using the tail length and the tail percent. The results of these values were statistically almost the same as the results of the tail moment (data are not shown). These results suggest that 24 h exposure to 60 GHz millimeter-wavelength radiation may have no significant effect on the comet assay of HCE-T and SRA01/04 cells.

### 3.3. Hsp Expression

**Figure 4:**
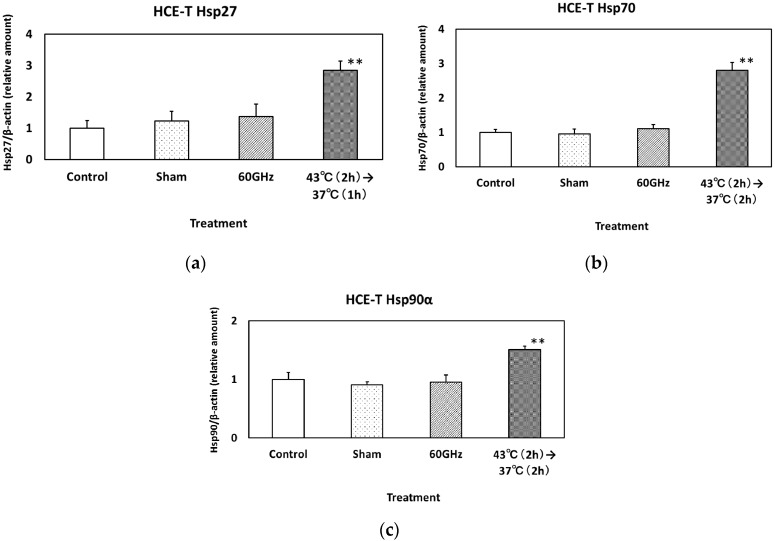
The expression of Hsp27 (**a**); Hsp70 (**b**) and Hsp90α (**c**) in HCE-T cells exposed to 60 GHz radiation for 24 h. The positive control underwent heat treatment at 43 °C for 2 h. Data are presented as the mean ± SD from three independent experiments. Asterisks ** indicate *p* < 0.01.

**Figure 5:**
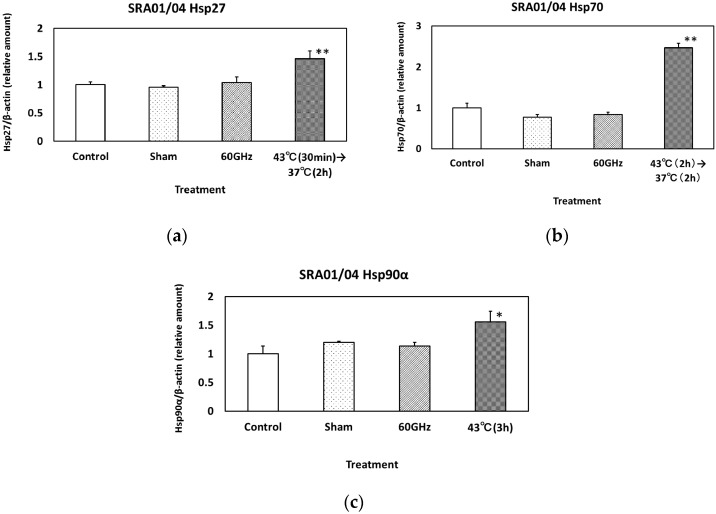
The expression of Hsp27 (**a**); Hsp70 (**b**) and Hsp90α (**c**) in SRA01/04 cells exposed to 60 GHz radiation for 24 h. The positive control underwent heat treatment at 43 °C for 30 min (Hsp27), 2 h (Hsp70) and 3 h (Hsp90α). Data are presented as the mean ± SD from three independent experiments. Asterisks * and ** indicate *p* < 0.05, and 0.01, respectively.

The expression of Hsp27, 70 and 90α in HCE-T cells is shown in Figure 4a–c, respectively. Heat treatment (Hsp27: 43 °C (2 h) to 37 °C (1 h), Hsp70 and 90α: 43 °C (2 h) to 37 °C (2 h)) clearly increased the level of each Hsp. The expression of Hsp27, 70 and 90α in SRA01/04 cells is shown in Figure 5a–c, respectively. Heat treatment (Hsp27: 43 °C (30 min) to 37 °C (2 h), Hsp70: 43 °C (2 h) to 37 °C (2 h), Hsp90α: 43 °C (3 h)) significantly increased the level of each Hsp.

## 4. Discussion

In this study we evaluated the effects of exposure to 60 GHz millimeter-wavelength radiation at a constant temperature for 24 h on cellular genotoxicity and stress responses using HCE-T and SRA01/04 cells. No effects were detected on MN formation, single-strand breaks in the DNA, and the expression of Hsp27, 70 and 90α, indicating the absence of non-thermal effects of millimeter-wavelength exposure. 

Several studies have evaluated the biological effects of millimeter-wavelength exposure and found effects on neuronal activity [11], proliferation [19], and cell metabolism [20], as well as effects on genomic instability [2]. Korenstein-Ilan et al. described that the continuous application of 0.1 THz radiation (0.031 mW/cm^2^) to human lymphocytes for 2 or 24 h induced genomic instability of chromosomes 11 and 17, detected using a fluorescence in situ hybridization method, and suggested that such exposure may result in an increased risk of cancer. In contrast, in the present study, we only detected micronucleus formation (Figure 2) and DNA strand breaks (Figure 3) induced by bleomycin treatment, and not by exposure to 60 GHz millimeter-wavelength radiation for 24 h, suggesting that exposure did not cause an increase in genotoxicity. These contradictory results may be due to differences in the frequency, power density or experimental design between the two studies, and thus the underlying factors for these discrepancies require further investigation. 

Alexandrov et al. reported data showing non-thermal effects on the gene expression of cells following exposure to terahertz radiation [3]. They used mesenchymal mouse stem cells exposed to broadband 10 THz pulsed radiation, a system that is totally different from that used in the current study. Importantly, they did not detect differences in the protein levels of heat shock proteins Hsp105, 90 and CPR, consistent with our study, although the expression levels of the three corresponding genes showed clear effects of THz irradiation. In our study, we detected an increase in Hsp expression following heat treatment, used as a positive control, but not by exposure to 60 GHz millimeter-wavelength radiation for 24 h, suggesting that this exposure did not cause upregulation of Hsp. Further work is required to investigate other proteins in addition to the Hsp27, 70, and 90α explored in the current study.

A large body of literature reports no adverse effects of exposure to millimeter-wavelength radiation. Beneduci et al. [21] showed that long-term exposure of human skin melanoma cells to 42 GHz and 53 GHz radiation at a power density below 0.3 mW/cm^2^ did not induce any effect on the cell cycle of these cells. Nicolaz et al. [22] described that exposure to 60.4 GHz radiation at a power density of 0.14 mW/cm^2^ did not cause endoplasmic reticulum stress in human glial cells. Le Dréan’s group has also published studies on the effects of 60.4 GHz radiation [23,24,25]. Le Quément et al. [23,24] showed no significant differences in gene expression following exposure to millimeter-wavelength radiation: although after 6 h exposure at 20 mW/cm^2^ real-time PCR analysis showed that some gene expression levels were affected, and this effect was linked to the increased temperature caused by exposure. Haas et al. [25] also indicated that a slight increase in protein expression observed following exposure to 60.4 GHz for 24 h was related to heating, and that there were no differences in the protein expression of neuronal marker β-tubulin or in the internal expression of control β-tubulin, consistent with our data. However, in a separate study the same group proposed specific millimeter-wavelength effects [26]: specifically, that millimeter-wavelength exposure induced a drastic modification of the whole gene expression system primarily associated with the thermal effects of radiation, although no significant differences were found in the expression levels of several genes in the absence of thermal effects. We could not detect any effects on genotoxicity and Hsp expression in the current study, but the molecular mechanisms underlying cellular responses after millimeter-wavelength exposure remain to be determined.

Vijayalaxmi et al. [27] reported that genotoxicity tests provided no evidence for the induction of MN formation in murine peripheral blood and bone marrow cells exposed to 42 GHz electromagnetic millimeter-wavelength radiation, consistent with our data. Taken together, most studies to date have shown no effect of exposure to millimeter-wavelength radiation, although a few studies have shown effects. These controversial results may come from differences in the experimental conditions. Overall, it appears that exposure to millimeter-wavelength radiation has no genotoxicity effect, and does not alter Hsp expression in the absence of thermal effects. However, our study was performed on specific conditions. It has been shown that the effects of microwaves including millimeter-wavelength radiation strongly depend on a number of physical parameters such as frequency, modulation, polarization, background extremely low-frequency and static magnetic fields [28,29]. We have to be more careful in comparing the data which were performed at different conditions. In addition, we have to consider rigid statistical calculations which we might be missing. In this study, we obtained high statistical power in the MN test; however, we could not obtain enough statistical power in the comet assay. We should be carefully aware of these statistical issues.

We are currently conducting similar studies using 40 GHz radiation.

## 5. Conclusions

The findings from the present study suggest that exposure of HCE-T and SRA01/04 cells to millimeter-wavelength radiation at 60 GHz for 24 h has no significant effect on MN frequency, single-strand breaks in the DNA, or Hsp expression. In conclusion, the exposure of cells to millimeter-wavelength radiation at 60 GHz does not seem to have adverse effects on the genotoxicity or Hsp expression of cultured HCE-T and SRA01/04 cells using our specific experimental conditions, although the possible effects of other frequencies require further study.

## Abbreviations

The following abbreviations are used in this manuscript:

GHz	Gigahertz
THz	Terahertz
HCE-T	Human corneal epithelial
SRA01/04	Human lens epithelial
MN	Micronucleus
Hsp	Heat shock protein
